# An Investigation of the Knowledge, Attitudes, and Practices of Physicians Regarding Child Oral Health at Primary Health Centers in Qatar: A Cross-Sectional Study

**DOI:** 10.1055/s-0042-1743157

**Published:** 2022-09-05

**Authors:** Hadeel Mohammad Al-Qatami, Aisha Saleh Al-Jaber, Feras Hasan Abed Al Jawad

**Affiliations:** 1 Pediatric Dentistry Section, Hamad Dental Center, Hamad Medical Corporation, Doha, Qatar; 2 Orthodontics Section, Hamad Dental Center, Hamad Medical Corporation, Doha, Qatar

**Keywords:** oral health, questionnaire, knowledge, attitudes, practices, pediatricians, family physicians

## Abstract

**Objectives** The aim of this study was to investigate physicians' knowledge, attitudes, and practices (KAP) in relation to oral health in children attending baby well clinics in primary health care centers (PHCC) in Qatar.

**Materials and Methods** A cross-sectional design was adopted in which a piloted self-administered questionnaire was sent electronically to 417 physicians (pediatricians/family physicians) practicing in all PHCCs in Qatar. The questionnaire gathered information concerning demographic characteristics and KAP in relation to oral health in children. A score for each domain was given based on the percentage of correct answers.

**Statistical Analysis** Descriptive and analytical statistics were employed. For descriptive statistics, the frequency of distribution in relation to demographic data and responses to items of the questionnaire was presented. For analytical statistics, associations between independent variables (predictors) and KAP were assessed by employing univariate and multivariate logistic regressions. Predictors which were significantly associated in the unadjusted regression were entered into a final multivariate logistic regression to evaluate their effects after adjustment.

**Results** The response rate was 24%. The overall mean scores of KAP were 61, 60, and 44.4%, respectively. Females were more likely to give positive answers than males (odds ratio [OR] = 12.3, 95% confidence interval [CI] 2.4–62.2, *p* = 0.02). Age groups 35 to 45 and >45 years had significantly more correct answers than <35 years age group (OR= 7.5, 95% CI = 1.1–56.6 and 9.2, 95% CI 1.2–44.6, respectively, *p* = 0.05). No significant associations were found between any of the independent variables with attitudes. With respect to practices, multivariate logistic regression revealed that specialty was significantly associated with practices. Pediatricians were more likely to adopt positive practices when compared with family medicine physicians (OR, 95% CI = 5.3 (1–25.6), *p* = 0.04).

**Conclusion** Although physicians demonstrated moderate levels of knowledge and attitudes, this did not reflect positively on their practices. The overall score of practices was considered poor (44.4%). Poor practices in our sample reflected an urgent need for actions and plans to improve this aspect and confirmed other findings which stated that acceptable levels of knowledge or attitudes do not necessarily translate into favorable practices.

## Introduction

The oral cavity is among the main focal points for the interaction of the body with the external environment. 1 This pivotal link can play a major role in shaping an individual's general health. Historically, the impact of oral diseases on general health and mortality has been attributed to inflammatory or nutritional pathways. For example, evidence suggests that periodontal disease, one of the most common oral diseases, may lead to cardiovascular and respiratory-related mortality, while tooth loss affects dietary intake which, in turn, will negatively affects key nutrients intake. 2 Dental caries, which remains a pandemic disease across developing countries, if left untreated in children will lead to negative long-term consequences related to pain and chewing difficulties which may affect growth and cognitive development. 3 Further progression of tooth decay without intervention can lead to premature tooth loss which, in addition to compromising chewing and esthetics, will affect the future developing permanent dentition. 3 Furthermore, oral health can affect an individual's psychological and social well-being with clear link between impaired oral health and negative impact on the quality of life. 3

Due to the fact that newly born infants are regularly examined during their first year of life by non-dental health professionals, recommendations started to emerge in the past two decades calling for actions to integrate oral health monitoring and promotion to the overall general health assessment of infants. The main objectives of these recommendations were to engage primary care physicians in oral health assessment, prevention, and intervention. This, in turn, will aid in achieving healthy future oral development and maintaining a disease-free oral cavity.

To ensure successful involvement of non-dental health care professionals and lay down a sound foundation for this paradigm shift in expanding their role in oral health prevention and monitoring, the work of Lewis et al 4 was among the first to offer several recommendations based on a national survey which investigated pediatricians' knowledge, attitudes, and practices (KAP) regarding oral health, and to determine willingness to incorporate fluoride varnish into their practices. These included the need for adequate training for pediatricians in oral health promotion, the need for guidelines on preventive dental care, and the availability of sufficient resources to pursue successfully oral health-related activities.

As a result, several initiatives were introduced to empower the role of pediatricians in providing oral health screening, preventive oral care, anticipatory guidance, fluoride varnish application, and referring to dentist when dental intervention is needed. 5 Despite all efforts to integrate oral health promotion and care in medical settings and to increase the awareness of medical physicians involved in infants' general health care monitoring, several reports revealed that the level of physicians' engagement and activities related to oral health monitoring and assessment is limited and requires further support. 6 A recent review concluded that pediatricians have limited knowledge and understanding in critical areas related to oral health such as identifying early signs of caries, recommended age for a first dental visit, the transmission of bacteria from mother to child, and knowledge about using fluorides. 7

In Qatar, early childhood caries remains one of the most prevalent public health problems with a prevalence of 89% in preschool children aged 4 to 5 years old. 8 This necessitates urgent action from decision-makers to promote preventive programs and raise the awareness level of the public toward the importance of oral health. Pediatric/family medicine physicians are no exception in their vital role. A recent study assessed the KAP of health professionals in Qatar toward oral health and found that despite demonstrating a positive attitude toward the anticipatory guidance elements of oral health, the knowledge of health care professionals on childhood oral health is rather limited. However, one of the main limitations of this study was the majority of participants were nurses (77.3%) with a small number of pediatrician/family medicine physicians which made the assessment of their KAP incomplete and unrepresentative. 9 No study evaluated KAP of only physicians who are involved in general health care assessment of children under 4 years old in Qatar. Such an assessment is beneficial and would provide an insight about whether educational programs and training are needed to empower the role of medical physicians in oral health promotion and prevention of oral diseases. Therefore, the aim of this study was to evaluate the levels of KAP of pediatricians/family physicians practicing in all primary health care centers (PHCC) in Qatar toward oral health.

## Material and Methods

For transparent reporting, this study was presented in accordance with the Strengthening the Reporting of Observational Studies in Epidemiology statement. 10

### Study Design

This was a prospective, cross-sectional study. Ethical approval was obtained from the Medical Research Center, Hamad Medical Corporation, proposal ID MRC-01-19–163, and Department of Clinical Research, Primary Health Care Corporation, Doha-Qatar (project number: PHCC/DCR/2019/10/030).

### Participants and Setting

The study population was physicians (family medicine and pediatricians) who were working in well-baby clinics (WBC) in all PHCC in Doha, Qatar. The WBC provides medical services based on international standards to all children under the 5 years of age. Physicians were identified from the database which was provided from the operations directorate office from which emails of physicians who provide health care in all WBC were retrieved. Only physicians whose primary role involved health care provision for children aged 5 years or below were included in the study. Nonspeaking English language physicians were excluded. Participation was voluntarily, and each physician was sent an information sheet describing the objectives of the study.

### Outcomes

The primary outcomes of this study were levels of physicians' KAP. Demographic variables were considered predictors (independent variables) which included gender, age, specialty, and experience years.

### Questionnaire

A structured self-administered questionnaire was used in the present study. Items of the questionnaire were adopted after a comprehensive literature review and based on the American Academy of Pediatric Dentistry (AAPD) recommendations for oral health assessment in infants. Modifications related to the number of items and response options were made. To ensure that items of the questionnaire were feasible and had good content validity, it was given to a group of five pediatric dentists and five pediatricians. Except for few wording amendments, no issues were reported in completing the questionnaire.

Construct validity could not be tested in relation to the outcomes of interest as they were not psychosocial constructs in which psychometric properties need to be tested. Furthermore, all studies that evaluated parents' KAP did not report on the construct validity and used different wording or options for items.

The final version of the questionnaire comprised of four domains.

The first domain assessed demographic data which included gender, age groups (categorized into ≤35, >35–45, and >45 years), years of experience (categorized into 0–10, 10–20, and >20 years), and specialty (family medicine or pediatrician).

The second domain assessed the knowledge of parents using eight items which covered knowledge about causative factors of dental caries, night feeding, frequency of sugar intake, when children should start brushing, fluoride, the time for the first dental visit, and whether caries is transmissible from the mother. Options for each item ranged from “yes,” “no” and “I don't know,” where “yes” corresponds to a positive knowledge to specific options relevant to a particular question.

The third domain assessed attitude (four items) which covered the importance of baby well clinics (BWC) in oral health assessment, obstacles to provide better oral health advice, the role of pediatricians in screening the oral cavity, and interest in obtaining educational training. Options for each item were “yes,” “no” and “I don't know,” where “yes” corresponds to a positive attitude.

The fourth domain assessed practice using five items which covered practices related to examining the oral cavity, asking parents whether they supervise their children when brushing their teeth, first dental visit, content of toothpaste, and sugar-free medications. Options for each item were similar to attitudes domain.

The questionnaire was generated via the online program Google Forms. A link was sent to all participants that included the questionnaire and an information sheet describing the aims of the study.

Data collection was done by the principal investigator (H.M.A.Q.) who adopted a standardized protocol with respect to sending emails. A reminder was sent to the physician if no reply was received. Data collection was performed between February and April 2020.

### Bias

A standardized protocol was adopted in sending emails to all physicians. In addition, data entry and analysis were done by independent persons who were not involved in the study.

### Sample Size Calculation

No sample size calculation was done. However, to ensure a good study power, all physicians working in BWC were approached in all the 27 PHCCs.

### Statistical Analysis

Descriptive and analytical statistics were employed. For descriptive statistics, the frequency of distribution in relation to demographic data and responses to items of the questionnaire were presented. For analytical statistics, associations between independent variables (predictors) and KAP were assessed by employing univariate and multivariate logistic regressions. Predictors which were significantly associated in the unadjusted regression were entered into a final multivariate logistic regression to evaluate their effects after adjustment. The scoring of KAP domains was based on the percentage of the correct answers (favorable answers). The response “yes” was considered a correct answer, whereas responses “no” or “I don't know” were considered incorrect answers. The percentage of correct answers for each domain was calculated by dividing the number of correct answers to the maximum possible number of correct answers multiplied by 100. A percentage of 49 or below was considered poor, 50 to 69 fair, and ≥70 good. However, to facilitate the regression analyses, the outcomes were dichotomized to either favorable (≥ 50%) or unfavorable answers (<50%). The *p* -value was set as 0.05, and SPSS software (version 22) was used for analysis.

## Results

A total of 417 questionnaires were electronically sent. Ninety-nine physicians completed and returned the surveys giving a response rate of 23.7%. Of the 99, 51 (51.5%) were females. The majority of respondents were family medicine physicians 88 (89%), and only 11 (11%) were pediatricians. The mean age of physicians was 38.5 years, and the majority had 0 to 10 years of experience ( Table 1 ).

**Table 1:** Demographic characteristics of the sample

Frequency		
Age (y)	Age ≤35 y	21
	Age >35 to 45 y	51
	Age >45 y	26
Number of years in practice	Year of experiences (0–10 y)	22
	Year of experiences (>10 to 20 y)	48
	Year of experiences (>20 y)	28
Specialty	Family medicine	87
	Pediatrician	11
Gender	Female	51
	Male	48

## Knowledge

The overall mean score of knowledge was 61%. The knowledge of 19.2% was good, 61.6% fair, and 19.2% poor ( Table 2 ).

**Table 2:** Frequency of KAP levels ( *n* = 99)

Valid	Poor *n* (%)	Fair	Good	Mean score
Knowledge	19(19.2)	61(61.6)	19(19.2)	61%
Attitude	14(14.1)	31(31.3)	54(54.5)	60%
Practice	61(61.6)	15(15.2)	23(23.3)	44.4%

Abbreviation: KAP, knowledge, attitudes, and practices.

Univariate logistic regression showed that gender, age groups, and years of experience were significantly associated with knowledge ( *p* = 0.02, *p* = 0.05, and *p* = 0.04, respectively) ( Table 3 ).

**Table 3:** Association between demographic data with knowledge score (categorized as >50% questions are correctly answered)

Predictor variables	Univariate logistic regression			Multivariate logistic regression	
	Percentage (%) of positive knowledge score	Unadjusted odds ratio (OR) and 95% CI	*p* -Value	Adjusted odds ratio (OR) and 95% CI	*p* -Value
Gender Male Female	66.7 96	1.0 (reference) 12 (2.6–55.6)	0.002	1.0 (reference) 12.3 (2.4–62.2)	0.002
Age group					
≤35 y	63.3	1.0 (reference)		1.0 (reference)	
> 35–45 y	84.3	3 (1–9.8)	0.05	7.5 (1–58.6)	0.05
> 45 y	88.5	4.4 (1–19.3)	0.05	9.2(1–44.6)	0.05
Years of experiences					
0–10 y	69.6	1.0 (reference)		1.0 (reference)	
> 10–20 y	79.2	1.6 (0.5–5.1)	0.38	0.4 (0.05–3)	0.358
> 20 y	93	5.7 (1–31)	0.04	24 (0.8–68.3)	0.999
Specialty					
Family medicine	79.5	1.0 (reference)		1.0 (reference)	
Pediatrician	91	2.5 (0.3–21.4)	0.38	1.1 (0.1–13.1)	0.897

Abbreviation: CI, confidence interval.

However, when the independent variables were entered into a multivariate logistic regression model, the independent variables, gender and age groups, remained significantly associated with knowledge ( *p* = 0.02 and 0.05, respectively; Table 3 ). Females were more likely to give positive answers than males (odds ratio [OR] = 12.3, 95% confidence interval [CI] 2.4–62.2). Age groups 35 to 45 and >45 years were likely to give more correct answers than the <35 years age group (OR= 7.5, 95% CI = 1.1–56.6 and 9.2, 95% CI 1.2–44.6, respectively).

Knowledge items with the highest percentages of favorable answers included the importance of fluoride, time, bacteria, sugar intake, and saliva in the process of dental caries. Items with the least percentages of favorable answers included whether amount or frequency is important in the process of caries in which 76% gave incorrect answer, 60% did not know when parents should start using fluoridated toothpaste, and 84% of participants did not know that dental caries can be transmitted from the mother.

## Attitudes

The overall mean score of attitudes was 60%. It was good in 55.4%, fair in 31.3%, and poor in 14.1% ( Table 2 ).

No significant associations were found between any of the independent variables and attitudes ( Table 4 ).

**Table 4:** Association between demographic data and attitudes scores (categorized as ≥50% questions are correctly answered)

Predictor variables	Univariate logistic regression			Multivariate logistic regression	
	Percentage (%) of positive knowledge score	Unadjusted odds ratio (OR) and 95% CI	*p* -Value	Adjusted odds ratio (OR) and 95% CI	*p* -Value
Gender Male Female	83.3 88	1.0 (reference) 1.4 (0.4–4.5)	0.511	1.0 (reference) 1.4 (0.4–5.1)	0.58
Age group					
≤35 y	77.3	1.0 (reference)		1.0 (reference)	
> 35–45 y	84.3	1.6 (0.4–5.5)	0.473	0.7 (0.1–4.3)	0.733
> 45 y	96.2	7.3 (0.7–68.6)	0.08	8.2 (0.2–78.7)	0.240
Years of experiences					
0–10 y	74	1.0 (reference)		1.0 (reference)	
> 10–20 y	87.5	2.4 (0.6–8.7)	0.161	3.3 (0.5–20.5)	0.187
> 20 y	93	4.5 (0.8–25.4)	0.081	1.3 (0.05–37.6)	0.850
Specialty					
Family medicine	86.4	1.0 (reference)		1.0 (reference)	
Pediatrician	82	0.7 (0.1–3.7)	0.685	0.3 (0.04–3.1)	0.348

Abbreviation: CI, confidence interval.

Items for attitudes with the highest percentages of favorable answers included that the WBC is a suitable venue to provide parents with dental advice (66%), their role in conducting clinical examinations (81.4%), and interest in obtaining educational training on oral health advice (88.2%).

## Practices

The overall mean score of practices was 44.4%. Physicians' practices were good in 23.3%, fair in 15.2%, and bad in 61.6% ( Table 2 ).

Univariate and multivariate logistic regressions revealed that specialty was significantly associated with practices ( Table 5 ). Pediatricians were more likely to give correct answers than family medicine physicians (OR= 5.3, 95% CI 1–25.6, *p* = 0.04).

**Table 5:** Association between demographic data and practice scores(categorized as ≥50% questions are correctly answered)

Predictor variables	Univariate logistic regression			Multivariate logistic regression	
	Percentage (%) of positive knowledge score	Unadjusted odds ratio (OR) and 95% CI	*p* -Value	Adjusted odds ratio (OR) and 95% CI	*p* -Value
Gender Male Female	31.3 46	1.0 (reference) 1.8 (0.8–4.2)	0.163	1.0 (reference) 2.1 (0.9–5.3)	0.080
Age group					
≤35 y	41	1.0 (reference)		1.0 (reference)	
> 35–45 y	35.3	0.8 (0.3–2.2)	0.649	0.9 (0.2–5)	0.980
> 45 y	42.3	1.1 (0.3–3.3)	0.992	7 (0.4–120)	0.170
Years of experiences					
0–10 y	39	1.0 (reference)		1.0 (reference)	
> 10–20 y	37.5	0.9 (0.3–2.6)	0.895	0.7 (0.1–3.5)	0.66
> 20 y	39.3	1 (0.3–3.1)	0.991	0.1 (0.04–1.5)	0.1
Specialty					
Family medicine	35.2	1.0 (reference)		1.0 (reference)	
Pediatrician	63.2	3.2 (0.8–12)	**0.05**	5.3 (1–25.6)	**0.04**

Abbreviation: CI, confidence interval.

Items with the most favorable answers included examining children's oral cavities and giving advice to parents to supervise their children's brushing. Items with the least correct answers included advice given to parents on when to have the first dental visit (55%) and the use of fluoridated toothpaste (60%).

## Discussion

Equally to dentists, medical professionals can play a major in preventing oral diseases and oral health promotion. In 2003, the American Academy of Pediatrics (AAP) published a policy statement which suggested that “pediatricians and pediatric health care professionals should develop the knowledge base to perform oral health risk assessments on all patients beginning at 6 months of age.” 11 Medical professionals should have adequate KAP regarding oral health issues to ensure their competency. Therefore, this study aimed to assess the KAP of pediatricians and family medicine physicians who are providing medical care in WBC in Qatar.

In this study, the overall knowledge was considered fair (61%). This finding was similar to other reports. 12 13 14 However, other studies reported suboptimal knowledge levels such as the study of Sabbagh et al which found that only 1.4% of pediatricians had scores higher than 60%. 15 Similarly, another study in the United States found that only 9% of pediatricians answered the knowledge questions correctly. 16 In both studies, it was concluded that pediatricians' knowledge regarding oral health issues such as identifying oral diseases, timing of primary teeth eruption, preventive measures, and referrals to dental professionals should be improved.

The participants in this study demonstrated areas of strengths with respect to their knowledge. For example, the majority acknowledged the importance of fluoride, time, bacteria, sugar intake, and saliva in the process of dental caries. This finding was similar to other studies. 7 Nevertheless, the physicians showed some areas of weaknesses that necessitated a high need for action. For example, most physicians (75%) answered that the amount of sugar intake resulted in more occurrence of dental caries. 14 It is well known that the frequency of sugar intake is as important as the amount in increasing the risk of caries; thus, medical physicians who are dealing with children should focus their dietary advice on reducing the frequency of sugar intake. 17 Another disappointing finding was the fact that most physicians (60%) did not know when parents should start using fluoridated toothpaste. This finding was in accordance with another study conducted in Saudi Arabia (KSA) in a group of pediatricians and family medicine physicians. 15 According to the AAP, it is recommended to use a “smear” of fluoride toothpaste twice a day when the first tooth appears and until age 3 years. 18 As such, the knowledge of pediatricians regarding the benefits of fluoride and the doses required needs reinforcement and improvement as the key areas.

Unfortunately, 84% of participants did not know that dental caries can be transmitted between a mother and her child. This indicated that there is a need to reinforce the knowledge of physicians with respect to caries etiology including modes of transmissions. 19

Knowledge of participants was significantly associated with gender, age, and years of experience. However, when the variables were entered into the regression model, the significance of years of experience was not confirmed yielding only gender and age as significant predictors. Females were significantly more knowledgeable than males which was in agreement with several studies. 20 21 22 23 This finding could be attributed to the fact that a considerable number of female physicians in this study might be mothers (no data on the number of physicians who are mothers). It is well known that mothers are generally more involved in the daily care of their children including oral hygiene care. In contrast, Rabie et al found that males scored higher knowledge scores. 24 Based on our finding, infants seen in the BWC might not be receiving adequate dental assessment by male physicians which is an area that needs to be addressed.

Age was also a significant predictor. Physicians who were above 45 years old demonstrated significantly better knowledge compared with younger clinicians. This finding appears to be logical as older physicians accumulate a great deal of experience, skills, and knowledge.

With respect to attitudes, the study showed that, overall, it was also fair (60%). Only 14% demonstrated poor attitudes, which was an encouraging finding. This agreed with the findings of Bhoopathi et al 14 who found that most of the physicians had favorable attitudes toward oral health issues. Among areas of positive attitudes that were demonstrated, most participants believed that the WBC is a suitable venue to provide parents dental advice and pursue regular check-ups for their children. Moreover, most participants showed positive attitudes with respect to their roles in preventing oral diseases and conducting clinical examinations. This was consistent with other studies. 7 It was an encouraging finding that most participants would be interested in obtaining training on delivering oral health advice to parents, meaning positive attitudes and openness toward updating their current knowledge in aspects related to their clinical roles including oral health care. In a survey conducted in the United States on 854 pediatricians, it was found that pediatricians overwhelmingly believed that they play an important role and are already involved in providing anticipatory guidance on oral health issues. 20 However, lack of up-to-date information and proper training were the main barriers in improving their levels of dental care. The survey recommended that formal training about oral health should be incorporated in medical schools along with continuous educational programs that should be organized regularly to reinforce their roles in oral health maintenance for young children.

Although participants demonstrated moderate levels of knowledge and attitudes, this did not reflect positively on their practices. The overall score of practices was considered poor (44.4%). This finding was in accordance with other surveys conducted in KSA. In contrast, another study in India showed that most of primary health care workers demonstrated higher levels of favorable practices (81%). 14 Poor practices in our sample reflected an urgent need for actions and plans to improve this aspect and confirmed other findings which stated that acceptable levels of knowledge or attitudes do not necessarily translate into favorable practices. 12

One of the most important findings of this study was the significant difference in practice scores between pediatricians and family medicine physicians ( *p* = 0.02).

Here, pediatricians showed higher levels of favorable practices. This finding indicated that there is a gap between the knowledge of family medicine physicians and their practices which as result requires urgent interventional educational programs to improve this aspect. The same result was found in other surveys. 12 It would also appear that acceptable levels of knowledge and attitudes are not enough to reflect on practices. Other factors might play a role such as lack of quality time during clinical examinations and the fact that parents have little interest in issues related to oral screening and monitoring.

Key practice deficits were identified in the present study. Areas that needed improvement were related to advice given to parents on when to attend the first dental visit and the use of fluoridated toothpaste. In both aspects, most of the participants gave unfavorable answers (55 and 60%, respectively). According to the AAPD, the child should visit the dentist within the 6 months of the eruption of the first primary tooth or by age of 1 year. Furthermore, dietary counseling and advice related to fluoride importance should be taught to parents during infants' well-clinics. Therefore, every effort should be made to reinforce the role of pediatricians and family medicine physicians in clinical settings and ensure that their practices conform to the recommended standards. 25 On a positive note, most participants reported that they examined children's oral cavity and they advised parents to supervise their children's brushing with fluoridated toothpaste.

The present study had several limitations. The sample might be unrepresentative as only physicians from PHCCs were recruited. Recruiting physicians from other sectors such as private practices could have improved the generalizability. In addition, the response rate was suboptimal which may increase the chance of respondents' bias.

This low response rate might be attributed to the timing at which the survey was conducted during the outbreak of coronavirus disease 2019 which might have overwhelmed the respondents. That is, physicians who did not respond might have different KAP, thus, affecting the findings of this study. Moreover, bias related to social desirability might have affected the outcomes as well. Finally, although face and content validity of the questionnaire used in the present study was assessed, it is unlikely that it captured all aspects of KAP. However, there is no standardized questionnaire to be used globally and there are variations in questionnaires used to assess KAP across studies. Additionally, the outcomes investigated were not psychosocial constructs that required psychometric characteristics assessment. They were items related to clinical practices that are based on standardized international recommendations. 11

## Conclusions

The participants demonstrated fair levels of knowledge and attitudes and poor levels of practices. Areas of strengths and weaknesses in KAP were identified. Female physicians were significantly more knowledgeable than males. Younger physicians need more support and educational programs to improve their knowledge with respect to oral health issues. When compared with pediatricians, family medicine physicians need to improve their practices at the clinical level by organizing regular training programs to empower their roles in oral health care promotion.
